# Efficacy of Cold-Stimulus Interventions for Thirst Management in Adult Postoperative Patients: A Systematic Review Protocol

**DOI:** 10.3390/nursrep16090322

**Published:** 2026-09-04

**Authors:** Ayano Ando, Runa Tokunaga, Takahiro Mihara, Makoto Kaneko

**Affiliations:** 1 Department of Nursing, Faculty of Medical Technology, Teikyo University, 2-11-1 Kaga, Itabashi-ku, Tokyo 173-8605, Japan; 2 Department of Health Data Science, Yokohama City University, Yokohama 236-0027, Japan; 3 Department of Nursing, Faculty of Human Sciences, Sophia University, Tokyo 102-8554, Japan; 4 Department of General Medicine, School of Medicine, Kitasato University, Sagamihara 252-0374, Japan

**Keywords:** postoperative thirst, cold stimulation, thirst management, randomized controlled trials

## Abstract

**Background/Objectives**: Postoperative thirst is a common and distressing symptom among surgical patients; however, standardized management guidelines remain limited. Oral care interventions using cold stimulation (e.g., ice chips, popsicles, and menthol-infused products) may relieve postoperative thirst, but variation in intervention protocols and outcome measures has so far precluded clear clinical recommendations. This protocol outlines a systematic review evaluating the effectiveness of cold-stimulation oral care interventions in reducing thirst intensity among adult surgical patients during the immediate postoperative period, defined as the first 6 h after emergence from general anesthesia. **Methods**: Randomized controlled trials (RCTs) comparing cold-stimulation oral care with standard care will be included; as pre-specified at registration, quasi-randomized trials will be considered only if fewer than three eligible RCTs are identified (appraised with ROBINS-I and reported separately). MEDLINE, Cochrane Library, EMBASE, CINAHL Plus and trial registries were searched. Risk of bias will be assessed using the Cochrane Risk of Bias tool version 2.0. The primary outcome is change in thirst intensity; secondary outcomes are oral dryness, patient satisfaction, and adverse events. Continuous outcomes will be pooled as mean differences, or as standardized mean differences across differing scales, and dichotomous outcomes as risk ratios, with 95% confidence intervals. Random-effects meta-analysis will be performed in R where appropriate, otherwise narrative synthesis, with pre-specified subgroup and sensitivity analyses. Certainty of evidence will be rated using GRADE. **Conclusions**: This protocol was developed in accordance with PRISMA-P and prospectively registered in PROSPERO (CRD420251139881). The review will clarify the effectiveness and safety of cold-stimulation oral care for postoperative thirst and support perioperative nursing practice.

## 1. Introduction

Postoperative thirst is a distressing perioperative symptom experienced by patients, and as frontline providers of perioperative care, nurses are responsible for recognizing and addressing such symptoms. Approximately 76.8% of patients have been reported to experience some degree of thirst during the immediate postoperative period [1], alongside nausea, vomiting and pain, as a major source of discomfort. Nevertheless, the assessment and management of postoperative thirst in clinical settings remain insufficiently systematized, and many nurses lack adequate recognition of this symptom and knowledge of how to respond to it [2,3].

The etiology of postoperative thirst is multifactorial, with prolonged preoperative fasting, effects of anesthetic agents, oral dryness related to endotracheal intubation, and intraoperative dehydration having been identified as key contributors [4]. Neurophysiologically, thirst is regulated by complex mechanisms centered in the hypothalamus, and thirst perception is influenced by arginine vasopressin secretion as well as osmoreceptor and baroreceptor activity [5].

In recent years, non-pharmacological interventions with cold stimulation have garnered considerable attention as strategies for alleviating postoperative thirst. Cold stimuli (e.g., ice chips, cold water, and menthol-based ice particles) provide an oral cooling sensation without actual fluid intake, thereby inducing a sense of comfort and reassurance [6]. These interventions are not only non-invasive and simple but also do not require specialized equipment or costly resources, making them highly feasible for immediate implementation in clinical practice [6]. However, existing studies are limited to individual clinical trials, and comprehensive evidence regarding the overall effectiveness of and optimal conditions for cold-stimulation interventions, such as the type of stimulus, timing, and duration, remains scarce.

Two evidence syntheses have previously addressed this topic: a scoping review by Silva et al., which maps existing literature but does not estimate intervention effects [6], and a systematic review of cold oral applications by Çelik et al., which searched five databases (not including the Cochrane Library, EMBASE, or CINAHL Plus) to January 2023, was restricted to English- and Turkish-language reports, and synthesized the results narratively without meta-analysis or a rating of the certainty of evidence [7]. This review is therefore intended not as a duplication but as an extension, in five specific respects: (i) The search covers each database from inception to 12 May 2026 with no language restriction and adds the Cochrane Library, EMBASE, and CINAHL Plus as well as clinical trial registries, thereby capturing trials published after the earlier search and reducing language and publication bias. (ii) Eligibility is restricted a priori to randomized designs so that a quantitative synthesis is interpretable. (iii) The outcome window is defined a priori as the immediate postoperative period (within 6 h after emergence from general anesthesia), whereas the earlier syntheses combined heterogeneous assessment windows. (iv) A meta-analysis with pre-specified effect measures, together with pre-specified subgroup analyses by type of cold stimulus, type of surgery, timing of assessment, and age, will be used to quantify effects and to explore the sources of heterogeneity that previous authors identified but could not resolve. (v) The certainty of evidence will be rated using the GRADE approach and presented in a Summary of Findings table.

Accordingly, this study protocol describes a systematic review aimed at evaluating the effectiveness of oral interventions with cold stimulation in reducing the thirst intensity in adult surgical patients during the immediate postoperative period, as compared with use of moistened gauze, humidification, or no intervention. This review is grounded in the recognition that postoperative thirst has been overlooked in clinical practice [2]. By clarifying the effectiveness and safety of a non-pharmacological and readily implementable intervention, the findings of this study may contribute to improving the quality of postoperative care. By synthesizing evidence from individual studies, this review may inform the development of standardized nursing guidelines and support consistency of care for adult patients in the immediate postoperative period, including those in post-anesthesia care units (PACUs) and general wards. Furthermore, building an evidence base for cold stimulation—a simple and low-cost intervention—holds substantial significance in expanding its applicability to diverse healthcare settings, including those with limited resources [7].

This protocol aims to describe a systematic review investigating whether oral care interventions with cold stimulation (e.g., ice chips, ice popsicles, or menthol-based products) are effective in reducing postoperative thirst in adult surgical patients during the immediate postoperative period (within 6 h after emergence from general anesthesia), as compared with standard care or non-cold oral interventions (e.g., moistened gauze or humidification).

## 2. Methods and Analysis

This systematic review and meta-analysis will follow the Cochrane Handbook for Systematic Reviews of Interventions [8]. The protocol is registered in the International Prospective Register of Systematic Reviews (PROSPERO; registration no.: CRD420251139881) and developed in accordance with the Preferred Reporting Items for Systematic Review and Meta-Analyses Protocols (PRISMA-P) 2015 checklist [9]. The protocol was originally registered while the corresponding author was affiliated with Sophia University; her current affiliation is that given above. Any amendment to this protocol, and any deviation from it that becomes necessary during the conduct of the review, will be recorded in the PROSPERO registration with the date and rationale, and will be reported transparently in the completed review in accordance with PRISMA 2020.

### 2.1. Patient and Public Involvement

No patients or members of the public were involved in the design, conduct, reporting, or dissemination plans of this protocol.

### 2.2. Eligibility Criteria

#### 2.2.1. Type of Studies

The primary eligibility criterion for study design is the randomized controlled trial (RCT). Non-randomized studies—including non-randomized comparative studies, uncontrolled before-and-after studies, case series, case reports, editorials, and reviews—will be excluded. As pre-specified in the PROSPERO registration, quasi-randomized trials (i.e., trials in which participants were allocated by a systematic but non-random method such as alternation, date of birth, or hospital record number) will be considered for inclusion only in the event that fewer than three eligible RCTs are identified for the primary outcome. Should that contingency arise, quasi-randomized trials will be appraised with the Risk Of Bias In Non-randomized Studies of Interventions (ROBINS-I) tool rather than with RoB 2 (Section 2.8), will not be pooled with RCTs in the primary analysis, and will be summarized separately and examined in a sensitivity analysis; the certainty of evidence contributed by this body of studies will be rated separately and will start at “low” in accordance with GRADE guidance for non-randomized evidence [10].

#### 2.2.2. Type of Participants

Adult surgical patients aged ≥ 18 years in the immediate postoperative period will be included. Throughout this protocol, the immediate postoperative period is defined as the first 6 h after emergence from general anesthesia; operationally, the start of this window is the time of tracheal extubation or, where this is not reported, the time at which recovery of consciousness was documented in the operating room or the post-anesthesia care unit (PACU). The same definition is used in the abstract, in the eligibility criteria, and in the outcome definitions. Where a trial reports the timing of outcome assessment only in relation to the end of surgery, that time point will be used as a proxy and the substitution will be recorded in the table of study characteristics. This 6-h cutoff was selected to focus on the early postoperative phase, during which thirst may be strongly influenced by perioperative factors such as fasting, anesthesia, endotracheal intubation, and surgical stress [2], and during which patients are typically still nil by mouth, so that cold stimulation of the oral cavity is one of the few available options; beyond this window, the resumption of oral fluid intake becomes the dominant determinant of thirst and would confound the comparison of interest. We acknowledge that this restriction will exclude trials that assessed thirst only at later time points, and we have accepted that loss of studies in exchange for a clinically coherent and mechanistically interpretable population.

All surgical procedures performed under general anesthesia will be eligible. This deliberately broad population was chosen for two reasons. First, the proposed mechanism of cold stimulation—cooling of the oral and oropharyngeal mucosa, which relieves the sensation of thirst without meaningful fluid intake—is not specific to any surgical specialty [6]. Second, the randomized evidence in this field is sparse, and restricting the population to a single procedure or specialty would leave too few trials for any meaningful synthesis. We recognize that the type and complexity of surgery, the duration of anesthesia, and fluid management strategies all influence postoperative thirst, and that this breadth therefore introduces clinical heterogeneity. This heterogeneity will be addressed in three ways: (i) Type of surgery, duration of surgery or anesthesia, and patient age are pre-specified as subgroup variables (Section 2.10). (ii) A random-effects model will be used throughout (Section 2.9). (iii) Where the eligible population is substantially broader than the clinical question of interest, the certainty of evidence will be downgraded for indirectness or inconsistency within the GRADE framework (Section 2.12).

*Eligibility of mixed populations.* Trials including both eligible and ineligible participants will be included if data for the eligible subgroup can be extracted separately. If data for the eligible subgroup cannot be obtained, the trial will be excluded.

#### 2.2.3. Types of Intervention

In this review, “intervention” refers to any oral care intervention involving cold stimulation intended to alleviate postoperative thirst. Eligible interventions may include ice chips, ice particles, ice popsicles, menthol-infused frozen products, chilled gauze, and other methods intended to provide a cooling sensation in the oral cavity. An intervention will be classified as “cold stimulation” when it satisfies at least one of two operational criteria: (i) a thermal criterion—application to the oral cavity or oropharynx explicitly described as frozen, iced, chilled, refrigerated, or cold; or (ii) a chemical criterion—content of menthol or another TRPM8 agonist, irrespective of delivery temperature. Interventions satisfying neither criterion will be treated as potential comparators.

These modalities are grouped under a single conceptual heading because they address the same clinical question: each is intended to prevent or relieve postoperative thirst, and each acts through the same putative mechanism—direct cooling of the oral and oropharyngeal mucosa, which relieves the sensation of thirst without meaningful fluid intake [6]. Because they share this common clinical purpose and this common mode of action, they were judged to be combinable within a single synthesis addressing that question. This does not imply that they are clinically equivalent in every respect: ice chips, frozen menthol-based products, chilled gauze or swabs, and cold sprays or rinses differ in temperature, volume, contact time, frequency of administration, and mode of delivery, and menthol-containing products additionally act on cold-sensitive receptors by a chemical route. These differences are treated as a potential source of statistical heterogeneity rather than as a reason to withhold synthesis. Accordingly, the type of cold stimulus (ice chips or ice particles; frozen products, including menthol-containing popsicles; chilled gauze or swabs; and cold sprays or rinses) is pre-specified as a subgroup variable, and where a sufficient number of trials contribute (Section 2.10), the extent to which these differences account for the observed heterogeneity will be explored in that analysis. Where substantial heterogeneity remains and the test for subgroup differences indicates that the modalities do not behave similarly, estimates by type of stimulus will be presented alongside the pooled estimate and the certainty of the pooled estimate will be downgraded for inconsistency.

Comparators may include usual care, room-temperature water, moistened gauze, no intervention, and other non-cold thirst-relieving interventions. Interventions not primarily based on cold stimulation, such as pharmacologic sialogogues or intravenous fluid administration, will not be considered eligible interventions.

*Handling of combined interventions.* Trials involving co-interventions will be included only when the co-interventions are applied equally to both groups, allowing the effect of cold stimulation to be evaluated separately. Trials in which the effect of cold stimulation cannot be isolated will be excluded.

If the classification of an intervention as “cold stimulation” is unclear, intervention details will be reviewed and, if necessary, the corresponding authors will be contacted for clarification. Final eligibility will be determined through discussion between two independent reviewers.

#### 2.2.4. Types of Outcome Measures

##### Primary Outcome

The primary outcome will be the change in postoperative thirst intensity during the immediate postoperative period, i.e., the change from the pre-intervention (baseline) assessment to the post-intervention assessment within 6 h after emergence from general anesthesia. Studies employing validated assessment scales such as the Numerical Rating Scale (NRS), Visual Analog Scale (VAS), Numerical Verbal Scale (NVS), Perioperative Thirst Discomfort Scale (PTDS), or the Thirst Symptom Assessment Scale (TSAS) will be included [11,12,13,14,15]. Trials assessing thirst only after the intervention, without a baseline measurement, will also be included. Final values will be analyzed as described in Section 2.9.

*Hierarchy and comparability of thirst measures.* These instruments are appropriate but are not interchangeable: NRS, VAS, and NVS are single-item scales that measure the intensity of thirst, whereas the PTDS and the TSAS are multi-item scales that measure the broader construct of thirst-related discomfort or symptom burden. We therefore pre-specify the following rules: (i) When a trial reports more than one eligible measure, a single measure will be extracted for the primary analysis using the hierarchy NRS > VAS > NVS > PTDS > TSAS; the measure actually used will be reported for every included study. (ii) Results from the single-item intensity scales (NRS, VAS, NVS) will be linearly rescaled to a common 0–10 metric and pooled as mean differences, because these scales measure the same construct on interchangeable metrics. (iii) The multi-item discomfort scales will not be rescaled onto the intensity metric; they will be analyzed as a separate outcome, and where trials measuring the two constructs must contribute to a single analysis, the standardized mean difference will be used instead. (iv) The choice of construct will also be examined in a sensitivity analysis restricted to trials that used single-item intensity scales.

##### Secondary Outcomes

Secondary outcomes will include improvement in oral dryness, patient satisfaction, and adverse events related to the interventions. Outcome measures may include validated oral dryness scales, salivary flow measures, satisfaction or comfort ratings, and adverse events such as aspiration, choking, allergic reactions, nausea or vomiting, and oral injury.

### 2.3. Exclusion Criteria

Studies will be excluded if:

1. The full text is unavailable despite the retrieval procedures described in Section 2.6, or sufficient data cannot be extracted despite the procedures for requesting missing information described in Section 2.7;
2. The record is a duplicate, or the publication is a secondary report of an already included trial that contains no additional usable data (Section 2.6 and Section 2.7);
3. The study design does not meet the eligibility criteria;
4. The study exclusively focuses on patients for whom thirst assessment or oral interventions are not feasible (e.g., prolonged ICU management, strict fasting orders, or severe impairment of consciousness).

### 2.4. Time Frame and Language

No restrictions will be applied regarding publication year or language. All studies published before the date of the final search will be considered.

### 2.5. Search Strategy

A comprehensive literature search was performed in MEDLINE (via PubMed), the Cochrane Library (via the Wiley online platform, including the Cochrane Central Register of Controlled Trials, CENTRAL), EMBASE (via Ovid), and CINAHL Plus (via EBSCOhost). These four databases were selected in consultation with a research librarian because together they provide near-complete coverage of the literature relevant to a nursing intervention question: MEDLINE and EMBASE for the biomedical and perioperative literature; CINAHL Plus for the nursing and allied health literature (including journals not indexed in MEDLINE); and CENTRAL, which aggregates trial records identified from multiple sources, including hand-searching and trial registries, for randomized trials. Multidisciplinary databases such as Web of Science and Scopus were not searched separately because their added value for a clinically focused question of this kind lies mainly in indexing journals already covered by the four databases above and in citation searching; to compensate, we searched clinical trial registries (the WHO International Clinical Trials Registry Platform, ClinicalTrials.gov, and the EU Clinical Trials Register) to identify ongoing, unpublished, or unreported studies, and we will additionally screen the reference lists of all included studies and of the existing reviews in this field [6,7], and contact the authors of the studies where necessary. No restrictions on publication date or language were applied, and all relevant studies published from database inception to the date of the search were considered.

The search strategy was developed in accordance with the Peer Review of Electronic Search Strategies (PRESS) statement [16], in consultation with a research librarian experienced in systematic review searching. Medical Subject Headings (MeSH) terms were used wherever possible and were combined with free-text terms in the title and abstract fields, with truncation applied as shown. The strategy deliberately combines a population block with an intervention block and does not require an outcome (thirst) term, so as to retain sensitivity: a version requiring thirst terms in the title or abstract was piloted during development and failed to retrieve trials known to be eligible, and it was therefore not adopted. A conceptual overview of the strategy is presented in Table 1, and complete search strategies for MEDLINE (via PubMed), EMBASE (via Ovid), the Cochrane Library (via Wiley), and CINAHL Plus (via EBSCOhost), including the database-specific translations of the PubMed strategy, are provided in Supplementary Table S1. Search terms used in the clinical trial registries are also reported there. The numbers of records retrieved and exported for screening were as follows: MEDLINE via PubMed, n = 1332; Cochrane Library, n = 1586; EMBASE, n = 2725; and CINAHL Plus, n = 265. The full flow of records, including the number of duplicates removed and the number of studies screened at each stage, will be reported in the PRISMA 2020 flow diagram of the completed review.

Status of the review at the time of the original submission: The protocol was registered in PROSPERO on 3 September 2025, and the final electronic search was executed on 12 May 2026. At the time of the original submission of this protocol, database searching and de-duplication had been completed and title-and-abstract screening was in progress. Full-text eligibility assessment, data extraction, risk-of-bias assessment, GRADE rating, and quantitative synthesis had not been started, and no effect estimate of any kind has been generated. The analysis plan reported here is therefore prospective with respect to the results of the review. For transparency, steps that have already been completed are described in the past tense and steps that have not yet been undertaken are described in the future tense throughout the Methods section.

### 2.6. Selection of Studies

Two reviewers will independently screen the studies based on the eligibility criteria. Titles and abstracts will be screened first, followed by full-text assessment of potentially eligible studies. Any disagreements between reviewers will be resolved through discussion with a third reviewer to reach consensus. Duplicate records will be identified with the automatic duplicate detection function of Rayyan (web version, accessed on 1 May 2026; Rayyan Systems Inc., Cambridge, MA, USA) and verified manually. Title-and-abstract and full-text screening will also be conducted in Rayyan [17]. The study selection process will be presented in a PRISMA 2020 flow diagram.

*Reports in languages other than English.* No report will be excluded based on language. Japanese reports will be assessed directly by the review team, and reports in other languages will be assessed using machine translation, with verification as needed.

*Unavailable full texts.* Full texts unavailable through institutional subscriptions will be sought through inter-library loan or by contacting the study authors. Records that remain unavailable will be excluded and documented in the PRISMA 2020 flow diagram.

### 2.7. Data Extraction and Management

Two reviewers will independently extract data using a standardized data extraction form. The data extraction form will be piloted on a small sample of included studies prior to full extraction to ensure consistency between reviewers. The following information will be extracted: author, publication year, country, study design, sample size, participant characteristics, type of surgery, intervention and comparator details, outcome measures, timing of outcome assessment, main findings, and adverse events. Any disagreements regarding data extraction will be resolved through discussion or consultation with a third reviewer.

*Handling of multiple reports of the same trial.* Multiple reports from the same trial will be identified and collated so that each trial is included only once. Data from all relevant reports will be combined without double counting.

*Handling of missing information.* When information relevant to eligibility, risk of bias, or effect estimation is missing, study authors will be contacted where necessary. Missing summary statistics will be derived from available reported data or handled as described in Section 2.9.

### 2.8. Assessment of Risk of Bias

Two independent reviewers will assess the risk of bias in the included RCTs using the Cochrane Risk of Bias tool version 2 (RoB 2) [8,18]. The assessment will include bias arising from the randomization process, deviations from intended interventions, missing outcome data, measurement of the outcome, and selection of the reported result, and each trial will be rated as being at low risk of bias, as having some concerns, or as being at high risk of bias for each domain and overall; the effect of assignment to the intervention (the intention-to-treat effect) will be the effect of interest. Only if the contingency described in Section 2.2.1 arises and quasi-randomized trials are included, those trials will be assessed with the ROBINS-I tool [10], which is designed for non-randomized studies of interventions, and their results will be reported separately from those of the RCTs. Any disagreements between reviewers will be resolved through discussion or consultation with a third reviewer.

### 2.9. Effect Measures, Unit of Analysis, Assessment of Heterogeneity, and Data Synthesis

*Effect measures.* For continuous outcomes, the effect measure will be the mean difference (MD) with a 95% confidence interval (CI) when all contributing trials use the same scale or scales that have been rescaled to a common metric (Section 2.2.4), and the standardized mean difference (SMD) with a 95% CI, calculated as Hedges′ g so as to correct for small-sample bias, when contributing trials use scales that measure the same construct on non-interchangeable metrics [8]. Because the primary outcome is defined as the change in thirst intensity, change-from-baseline scores will be used in preference to post-intervention values where both are reported. Trials reporting only post-intervention values will be included. For outcomes measured on the same scale or rescaled to a common metric, MDs based on change scores and final values will be combined in the same meta-analysis in accordance with the Cochrane Handbook [8]. Change scores and final values will not be combined within a single SMD analysis. Where the standard deviation of the change score is not reported, it will be derived from reported CIs, standard errors, or exact *p*-values, or, failing that, imputed using a correlation coefficient borrowed from another trial in the same analysis, with the impact of any imputation examined in a sensitivity analysis. For dichotomous outcomes—principally the incidence of adverse events and, where reported, the proportion of patients achieving a pre-defined reduction in thirst—the risk ratio (RR) with a 95% CI will be used, with the risk difference reported in addition when events are rare. All estimates will be interpreted in terms of both statistical and clinical relevance.

*Unit of analysis.* Each trial will contribute one estimate to any single meta-analysis. Consistent with the primary outcome as defined in Section 2.2.4—the change in thirst intensity from baseline to post-intervention—the pre-specified time point for the primary pooled analysis is, in each trial, the assessment performed after the first administration of the intervention within the 6 h window. Where a trial administers the intervention repeatedly or reports further assessments within that window, those later assessments will be analyzed as separate secondary analyses grouped by the timing categories defined in Section 2.10, so that no participant contributes more than once to any single pooled estimate. In multi-arm trials, only arms meeting the eligibility criteria will be included. Where two or more eligible cold-stimulation arms share a single control arm, those intervention arms will be combined into a single group for the primary analysis, so that each trial contributes one pair-wise comparison and no participant is counted twice; this is the approach recommended in the Cochrane Handbook and is consistent with the conceptual grouping of the cold-stimulation modalities set out in Section 2.2.3 [8]. As a sensitivity analysis, the comparisons will instead be kept separate, with the sample size of the shared control group divided approximately equally between them, and, where enough trials contribute, the individual arms will also be examined in the subgroup analysis by type of cold stimulus (Section 2.10).

*Assessment of heterogeneity and choice of model.* Clinical and methodological heterogeneity will be assessed before any pooling is attempted. Statistical heterogeneity will be assessed primarily using the I^2^ statistic, supplemented by the chi-squared (χ^2^) test and the between-study variance (τ^2^); substantial heterogeneity will be defined as I^2^ ≥ 50% or *p* < 0.10 for the χ^2^ test. A random-effects model will be used for all primary analyses. This choice is made a priori rather than conditionally on the observed heterogeneity because the eligible trials are expected to differ systematically in the type and dose of the cold stimulus, the type and duration of surgery, the anesthetic technique, and the timing of assessment, so that the assumption of a single common true effect underlying a fixed-effect model is not plausible; the random-effects model instead estimates the average of a distribution of effects and yields CIs that reflect this variability. Selecting the model on the basis of an observed I^2^ value would additionally make the analysis data-dependent, which we wish to avoid in a protocol. τ^2^ will be estimated using the restricted maximum likelihood (REML) estimator, and the CI for the pooled effect will be derived using the Hartung–Knapp–Sidik–Jonkman adjustment, which maintains nominal coverage when the number of contributing trials is small [19]. Fixed-effect estimates will be reported as a sensitivity analysis.

*Narrative synthesis.* If a meta-analysis is not considered appropriate—for example, because fewer than two trials report an outcome in a poolable form, or because clinical or methodological heterogeneity is judged too great to make an average effect meaningful—the findings will be synthesized narratively [8]. In that case, the effect estimate, its 95% CI, and the direction of effect will be tabulated for each study, results will be grouped by type of cold stimulus and by assessment time point, and no pooled estimate will be presented.

*Software.* All analyses will be performed in R (version 4.4.0 or later; R Foundation for Statistical Computing, Vienna, Austria) using the meta and metafor packages [20]. Summary of Findings tables will be prepared using GRADEpro GDT (web version; McMaster University and Evidence Prime Inc., Hamilton, ON, Canada; Section 2.12). Meta-regression is not planned: the number of eligible trials is unlikely to approach the minimum of approximately ten studies per covariate that is generally recommended, and any such analysis would therefore be uninformative [8].

### 2.10. Subgroup Analysis and Sensitivity Analysis

Subgroup analyses will be conducted for the primary outcome to explore potential sources of heterogeneity according to participant, intervention, and study characteristics. The following subgroups are pre-specified: age group (≥65 years vs. <65 years); sex; type of cold-stimulation intervention (ice chips or ice particles; frozen products, including menthol-containing popsicles; chilled gauze or swabs; cold sprays or rinses); type of surgery (e.g., abdominal, orthopedic, cardiothoracic, gynecological, other); duration of surgery or anesthesia; and timing of outcome assessment after the intervention. Timing of outcome assessment will be categorized as immediate (within 15 min), short-term (30–60 min), or prolonged (>1 h or repeated interventions). These windows were chosen pragmatically to mirror the assessment time points most commonly reported in the existing literature on oral cold applications [6,7] and because the relief produced by a single oral cold stimulus is short-lived, so that any effect still detectable beyond 1 h is likely to reflect repeated administration rather than the persistence of a single application; the cutoff is therefore pragmatic rather than derived from physiological data, and it will be applied consistently across trials.

Because we anticipate that only a small number of eligible trials will be identified, all subgroup analyses will be regarded as exploratory and hypothesis-generating, and their results will not be used to make claims about differential effectiveness. A subgroup analysis will be performed only when at least four trials contribute to the outcome overall and at least two trials fall within each category being compared; where these conditions are not met, the subgroups will be described narratively instead. Differences between subgroups will be assessed using a formal test for subgroup differences rather than by comparing *p*-values within subgroups, and the credibility of any apparent subgroup effect will be appraised qualitatively (number of comparisons made, whether the direction was pre-specified, and whether the effect is consistent across related outcomes). The number of subgroup analyses actually performed will be reported in full, including those that were pre-specified but could not be carried out.

Sensitivity analyses will be conducted to assess the robustness of the findings. The following are pre-specified: (i) exclusion of trials rated as being at high risk of bias overall with the RoB 2 tool; (ii) use of a fixed-effect model in place of the primary random-effects specification; (iii) exclusion of trials for which standard deviations of change scores had to be imputed; (iv) an alternative handling of multi-arm trials, splitting the shared control group between the separate comparisons rather than combining the eligible intervention arms (Section 2.9); and (v) restriction to trials that measured thirst with single-item intensity scales. Where the contingency described in Section 2.2.1 has led to the inclusion of quasi-randomized trials, an additional analysis excluding them will be presented. Participant characteristics and the timing of postoperative assessment will also be taken into account in the interpretation of the findings.

### 2.11. Assessment of Reporting Bias

For outcomes to which at least 10 studies contribute, funnel plots will be generated to assess asymmetry visually, and asymmetry will be examined statistically using Egger′s regression test [21] and Begg′s rank correlation test [22]. We emphasize, however, that both the visual and the statistical assessment of funnel-plot asymmetry have low power when few studies are available, and that these tests can yield misleading results when the contributing trials differ markedly in size or when between-study heterogeneity is substantial. Given the small body of randomized evidence anticipated in this field, we consider it likely that fewer than 10 trials will contribute to any single outcome; in that case, no formal test for funnel-plot asymmetry will be performed, no conclusion will be drawn from a visual inspection of the funnel plot alone, and the possibility of publication bias will instead be judged qualitatively within the GRADE framework. To support that judgment, clinical trial registries and other relevant sources will be screened to identify unpublished or unreported studies, and registered protocols identified in this way will be compared with the corresponding publications to assess selective outcome reporting.

### 2.12. Summary of Findings and Certainty of Evidence

For the primary and secondary outcomes, the certainty of evidence will be assessed using the GRADE approach [23], considering risk of bias, inconsistency, indirectness, imprecision, and publication bias. Outcomes including changes in thirst intensity, improvement in oral dryness, patient satisfaction, and adverse events will be evaluated. The certainty of evidence for each outcome will be rated as high, moderate, low, or very low. Evidence from RCTs will begin at high certainty; should the contingency described in Section 2.2.1 arise, evidence from quasi-randomized trials will be rated separately and will begin at low certainty. The reasons for any downgrading will be reported explicitly in a footnote to the table. The results of the assessment will be summarized in a Summary of Findings (SoF) table generated using GRADEpro GDT software.

## 3. Discussion

Postoperative thirst is one of the most distressing symptoms experienced by surgical patients, and reported prevalence varies across studies [1,24]. This symptom arises from a combination of factors, including preoperative fasting, effects of anesthetic agents, airway management, and physiological changes related to surgical stress. With the widespread adoption of enhanced recovery after surgery (ERAS) protocols, patients regain consciousness earlier and become more aware of their symptoms, which further underscores the importance of effective thirst management. Nonetheless, in current clinical practice, standardized guidelines for the management of postoperative thirst have yet to be fully established [2,25].

Cumulative evidence suggests that oral care interventions involving cold stimulation may be effective in alleviating postoperative thirst. However, existing studies vary considerably with respect to intervention protocols, measurement methods, target populations, and assessment timing, resulting in insufficient evidence to support clear recommendations for clinical practice. In a scoping review, Silva et al. highlighted the diversity of thirst management strategies in adult postoperative patients, underscoring the need for standardized intervention protocols [6]. Çelik et al. subsequently synthesized the evidence on cold oral applications narratively and reached broadly favorable but necessarily qualitative conclusions, as the included studies were not pooled and the certainty of the evidence was not formally rated [7]. The extent to which a quantitative synthesis is possible in this field therefore remains an open question, and it is one that the present review is designed to answer explicitly rather than to assume.

This systematic review will evaluate the effectiveness of cold-stimulation interventions during the immediate postoperative period and will attempt to identify the factors contributing to heterogeneity across studies. The extent to which it can strengthen the evidence base for nursing interventions in the management of postoperative thirst will depend on the number, size, and methodological quality of the trials identified. If a sufficient body of randomized evidence exists, the review could inform the development of clinical guidance for perioperative nursing; if it does not, the review will instead define the current limits of the evidence and specify the trials that would be needed to overcome them. In either case, we regard these as potential implications rather than as expected outcomes, and any recommendation for practice will be made only in proportion to the certainty of the evidence as rated by GRADE.

### Limitations

This review protocol has several limitations. First, the interventions grouped under the heading of cold stimulation differ in temperature, volume, contact time, and mode of delivery, and the eligible population spans all types of surgery performed under general anesthesia and a range of anesthetic and fluid management techniques; the resulting clinical heterogeneity may limit the interpretability of any pooled estimate, even though it will be explored through pre-specified subgroup analyses. Second, thirst is assessed with several instruments that are not interchangeable, ranging from single-item intensity scales to multi-item discomfort scales; the pre-specified hierarchy and rescaling rules (Section 2.2.4 and Section 2.9) make our approach transparent but rest on assumptions that may not hold for every trial. Third, the number of randomized controlled trials specifically addressing cold stimulation for thirst within the first 6 h after emergence from general anesthesia may be small; if it is, a meta-analysis may not be feasible for some or all outcomes, subgroup analyses will be underpowered, and formal tests for funnel-plot asymmetry will not be informative, so that the review may ultimately provide a structured narrative synthesis rather than pooled effect estimates. Fourth, participants cannot realistically be blinded to an oral cold stimulus and the primary outcome is patient-reported; risk of bias arising from deviations from intended interventions and from measurement of the outcome is therefore likely to be a recurrent concern and may lead to downgrading of the certainty of evidence. Fifth, the search was restricted to four databases and to trial registries, and grey literature such as conference abstracts, dissertations, and institutional reports was not systematically sought; some eligible studies may therefore not have been retrieved, and the four databases may under-represent regional nursing journals that are not indexed internationally. Finally, as this review depends entirely on existing studies, the methodological quality and the completeness of reporting of the primary research will inevitably constrain the strength of any conclusion that can be drawn.

## 4. Conclusions

This systematic review will comprehensively examine the effectiveness and safety of cold-stimulation oral care interventions during the immediate postoperative period. Depending on the quantity, quality, and consistency of the evidence identified, the findings may help to inform the development of standardized intervention protocols in nursing practice and to improve the management of postoperative thirst; where the available evidence proves insufficient, the review will instead clarify the nature of the gap and the design of the randomized trials needed to fill it. Because the interventions considered are simple and low-cost, any conclusions that can be drawn may be relevant across diverse clinical settings, including resource-limited environments, and may serve as a foundation for future research and practice.

## Figures and Tables

**Table 1:** Conceptual overview of the search strategy, developed in accordance with the PRESS statement (MEDLINE via PubMed). Terms listed within a block are combined with OR; blocks are combined as shown in the final line. The complete line-by-line strategy as executed in each database is provided in Supplementary Table S1.

Block	Search Terms (MEDLINE via PubMed)
#1 Population: surgery/operative procedure	“surgical procedures, operative”[MeSH Terms] OR “surgery”[tiab] OR “surgical”[tiab] OR “operative”[tiab] OR “operation*”[tiab]
#2a Cold stimulus (agent or vehicle)	“Ice”[MeSH Terms] OR “Ice”[tiab] OR “cold stimul*”[tiab] OR “Menthol”[MeSH Terms] OR “Mouthwashes”[MeSH Terms] OR “cooling”[tiab] OR “cooled”[tiab] OR “chilled”[tiab] OR “refrigerat*”[tiab] OR “iced”[tiab] OR “menthol*”[tiab] OR “mentholated”[tiab] OR “peppermint”[tiab] OR “TRPM8”[tiab] OR “cooling agent*”[tiab] OR “gargl*”[tiab] OR “spray”[tiab] OR “swab*”[tiab] OR “gauze”[tiab]
#2b Oral/oropharyngeal site	“oral”[tiab] OR “mouth”[tiab] OR “oropharyn*”[tiab] OR “throat”[tiab] OR “moisturis*”[tiab] OR “moisturiz*”[tiab] OR “lip hydrat*”[tiab]
#2c Named cold oral interventions	“ice chip*”[tiab] OR “ice pop*”[tiab] OR “popsicle*”[tiab] OR “mentholated popsicle*”[tiab] OR “ice lolly”[tiab] OR “ice lollies”[tiab] OR “ice cube*”[tiab] OR “ice water”[tiab] OR “iced drink*”[tiab] OR “cold drink*”[tiab] OR “cool water”[tiab] OR “oral cryotherapy”[tiab] OR “cold water spray”[tiab] OR “cold spray”[tiab] OR “mouth spray”[tiab] OR “throat spray”[tiab] OR “oropharyngeal moisturis* program*”[tiab] OR “cold water”[tiab] OR “wet gauze”[tiab] OR “moistened gauze”[tiab] OR “sponge swab*”[tiab] OR “lozenge*”[tiab] OR “mouthrinse*”[tiab] OR “mouth rinse*”[tiab] OR “crushed ice”[tiab] OR “slushie*”[tiab] OR “slushy*”[tiab]
#2 Intervention: cold oral/moisture intervention	(#2a AND #2b) OR #2c
#3 Study design filter (randomized trials, humans)	(“randomized controlled trial”[pt] OR “controlled clinical trial”[pt] OR randomized[tiab] OR randomised[tiab] OR placebo[tiab] OR “drug therapy”[sh] OR randomly[tiab] OR trial[tiab] OR groups[tiab]) NOT (animals[mh] NOT humans[mh])
#4 Final set	#1 AND #2 AND #3

Field tags: tiab = Title/Abstract; MeSH Terms = Medical Subject Headings; pt = publication type; sh = subheading; mh = MeSH heading. An asterisk (*) denotes truncation. All terms within a numbered block are combined with the Boolean operator OR unless otherwise indicated. The human filter is applied as NOT (animals[mh] NOT humans[mh]), i.e., records indexed as animal studies are excluded only when they are not also indexed as human studies.

## Data Availability

No new data were created or analyzed in this study. Data sharing is not applicable to this article, as this study is a systematic review protocol and all data will be obtained from published literature. The full search strategies as executed are provided in Supplementary Table S1.

## Supplementary Materials

The following supporting information can be downloaded at: https://www.mdpi.com/article/10.3390/nursrep16090322/s1. Table S1: Full search strategies for MEDLINE (via PubMed), EMBASE (via Ovid), the Cochrane Library (via Wiley), CINAHL Plus (via EBSCOhost), and the clinical trial registries; Table S2: PRISMA-P 2015 checklist.

## Public Involvement Statement

No public involvement in any aspect of this research was undertaken. No patients or members of the public were involved in the design, conduct, reporting, or dissemination plans of this protocol.

## Guidelines and Standards Statement

This protocol was developed in accordance with the Preferred Reporting Items for Systematic Review and Meta-Analysis Protocols (PRISMA-P) statement, and the completed PRISMA-P checklist is provided as Supplementary Material. The review is registered in PROSPERO (CRD420251139881).

## Abbreviations

The following abbreviations are used in this manuscript:

CENTRAL	Cochrane Central Register of Controlled Trials
CI	Confidence interval
ERAS	Enhanced recovery after surgery
GRADE	Grading of Recommendations Assessment, Development and Evaluation
ICTRP	International Clinical Trials Registry Platform
ICU	Intensive care unit
MD	Mean difference
MeSH	Medical Subject Headings
NRS	Numerical rating scale
NVS	Numerical verbal scale
PACU	Post-anesthesia care unit
PRESS	Peer Review of Electronic Search Strategies
PRISMA	Preferred Reporting Items for Systematic Reviews and Meta-Analyses
PRISMA-P	Preferred Reporting Items for Systematic Reviews and Meta-Analyses–Protocols
PTDS	Perioperative Thirst Discomfort Scale
RCTs	Randomized controlled trials
REML	Restricted maximum likelihood
RoB 2	Cochrane Risk of Bias Tool version 2.0
ROBINS-I	Risk Of Bias In Non-randomized Studies of Interventions
RR	Risk ratio
SMD	Standardized mean difference
SoF	Summary of findings
TSAS	Thirst Symptom Assessment Scale
VAS	Visual analog scale
