# Supplementary material for: Efficacy of Cold-Stimulus Interventions for Thirst Management in Adult Postoperative Patients: A Systematic Review Protocol
Source: Nurs Rep. 2026 Sep 4;16(9):322. doi: 10.3390/nursrep16090322 (PMC13610046; doi:10.3390/nursrep16090322)
Supplement: Supplementary file 1 [file nursrep-16-00322-s001.zip › nursrep-4451993-supplementary-final revised.pdf]

Supplementary Table S1. Full electronic search strategies as executed

Efficacy of Cold-Stimulus Interventions for Thirst Management in Adult Postoperative Patients: A Systematic Review Protocol

PROSPERO registration: CRD420251139881 | Date of final search: May 12, 2026

Platforms and interfaces: MEDLINE was searched via PubMed (National Library of Medicine); EMBASE via Ovid; the Cochrane Library (including CENTRAL) via the Wiley online platform; CINAHL Plus via EBSCOhost; and the trial registries via their public web interfaces (Section S1.6).

S1.1 MEDLINE (via PubMed) — searched May 12, 2026

The strategy below is reproduced exactly as executed. Terms within each block are combined with OR; blocks are combined as shown in lines #5, #7, and #8. An asterisk (*) denotes truncation. Field tags: [Title/Abstract] = title and abstract fields (tiab); [MeSH Terms] = Medical Subject Headings; [pt] = publication type; [sh] = subheading; [mh] = MeSH heading. Records retrieved and exported for screening: n = 1,332.

Line	Concept	Search string
#1	Population: surgery/operative procedure	("surgical procedures, operative"[MeSH Terms] OR "surgery"[Title/Abstract] OR "surgical"[Title/Abstract] OR "operative"[Title/Abstract] OR "operation*"[Title/Abstract])
#2	Cold stimulus (agent or vehicle)	("Ice"[MeSH Terms] OR "Ice"[Title/Abstract] OR "cold stimul*"[Title/Abstract] OR "Menthol"[MeSH Terms] OR "Mouthwashes"[MeSH Terms] OR "cooling"[Title/Abstract] OR "cooled"[Title/Abstract] OR "chilled"[Title/Abstract] OR "refrigerat*"[Title/Abstract] OR "iced"[Title/Abstract] OR "menthol*"[Title/Abstract] OR "mentholated"[Title/Abstract] OR "peppermint"[Title/Abstract] OR "TRPM8"[Title/Abstract] OR "cooling agent*"[Title/Abstract] OR "gargl*"[Title/Abstract] OR "spray"[Title/Abstract] OR "swab*"[Title/Abstract] OR "gauze"[Title/Abstract])
#3	Oral/oropharyngeal site	("oral"[Title/Abstract] OR "mouth"[Title/Abstract] OR "oropharyn*"[Title/Abstract] OR "throat"[Title/Abstract] OR "moisturis*"[Title/Abstract] OR "moisturiz*"[Title/Abstract] OR "lip hydrat*"[Title/Abstract])
#4	Named cold oral interventions (specific phrases)	("ice chip*"[Title/Abstract] OR "ice pop*"[Title/Abstract] OR "popsicle*"[Title/Abstract] OR "mentholated popsicle*"[Title/Abstract] OR "ice lolly"[Title/Abstract] OR "ice lollies"[Title/Abstract] OR "ice cube*"[Title/Abstract] OR "ice water"[Title/Abstract] OR "iced drink*"[Title/Abstract] OR "cold drink*"[Title/Abstract] OR "cool water"[Title/Abstract] OR "oral cryotherapy"[Title/Abstract] OR "cold water spray"[Title/Abstract] OR "cold spray"[Title/Abstract] OR "mouth spray"[Title/Abstract] OR "throat spray"[Title/Abstract] OR "oropharyngeal moisturis* program*"[Title/Abstract] OR "cold water"[Title/Abstract] OR "wet gauze"[Title/Abstract] OR "moistened gauze"[Title/Abstract] OR "sponge swab*"[Title/Abstract] OR "lozenge*"[Title/Abstract] OR "mouthrinse*"[Title/Abstract] OR "mouth rinse*"[Title/Abstract] OR "crushed ice"[Title/Abstract] OR "slushie*"[Title/Abstract] OR "slushy*"[Title/Abstract])
#5	Intervention set	(#2 AND #3) OR #4
#6	Randomized trial terms	("randomized controlled trial"[pt] OR "controlled clinical trial"[pt] OR randomized[Title/Abstract] OR randomised[Title/Abstract] OR placebo[Title/Abstract] OR "drug therapy"[sh] OR randomly[Title/Abstract] OR trial[Title/Abstract] OR groups[Title/Abstract])
#7	Human filter applied to #6	#6 NOT (animals[mh] NOT humans[mh])
#8	Final set	#1 AND #5 AND #7

Note on the number of records retrieved per line. This document accompanies a review protocol prepared in accordance with PRISMA-P 2015, which requires the information sources and a reproducible search strategy to be presented (items 9 and 10) but does not require the number of records returned by each line of that strategy. The PubMed search was executed as a single complete strategy, and the historical counts for each individual concept block were not set up as part of the search record required for a protocol. Because database indexing is updated continuously, re-running those lines now would return figures different from those of May 12, 2026, and we did not consider it accurate to present current counts as the results of that search. We therefore report the complete, reproducible strategy exactly as used, together with the total number of records actually exported for screening from each database at the time of the search (Section S1.8).

S1.2 Single-line version of the MEDLINE (PubMed) strategy

The following string reproduces line #8 and can be pasted directly into the PubMed search box.

```
("surgical procedures, operative"[MeSH Terms] OR "surgery"[Title/Abstract] OR
"surgical"[Title/Abstract] OR "operative"[Title/Abstract] OR "operation*"[Title/Abstract]) AND ( (
("Ice"[MeSH Terms] OR "Ice"[Title/Abstract] OR "cold stimul*"[Title/Abstract] OR "Menthol"[MeSH
Terms] OR "Mouthwashes"[MeSH Terms] OR "cooling"[Title/Abstract] OR "cooled"[Title/Abstract] OR
"chilled"[Title/Abstract] OR "refrigerat*"[Title/Abstract] OR "iced"[Title/Abstract] OR
"menthol*"[Title/Abstract] OR "mentholated"[Title/Abstract] OR "peppermint"[Title/Abstract] OR
"TRPM8"[Title/Abstract] OR "cooling agent*"[Title/Abstract] OR "gargl*"[Title/Abstract] OR
"spray"[Title/Abstract] OR "swab*"[Title/Abstract] OR "gauze"[Title/Abstract]) AND
("oral"[Title/Abstract] OR "mouth"[Title/Abstract] OR "oropharyn*"[Title/Abstract] OR
"throat"[Title/Abstract] OR "moisturis*"[Title/Abstract] OR "moisturiz*"[Title/Abstract] OR "lip
hydrat*"[Title/Abstract]) ) OR ("ice chip*"[Title/Abstract] OR "ice pop*"[Title/Abstract] OR
"popsicle*"[Title/Abstract] OR "mentholated popsicle*"[Title/Abstract] OR "ice lolly"[Title/Abstract]
OR "ice lollies"[Title/Abstract] OR "ice cube*"[Title/Abstract] OR "ice water"[Title/Abstract] OR
"iced drink*"[Title/Abstract] OR "cold drink*"[Title/Abstract] OR "cool water"[Title/Abstract] OR
"oral cryotherapy"[Title/Abstract] OR "cold water spray"[Title/Abstract] OR "cold
spray"[Title/Abstract] OR "mouth spray"[Title/Abstract] OR "throat spray"[Title/Abstract] OR
"oropharyngeal moisturis* program*"[Title/Abstract] OR "cold water"[Title/Abstract] OR "wet
gauze"[Title/Abstract] OR "moistened gauze"[Title/Abstract] OR "sponge swab*"[Title/Abstract] OR
"lozenge*"[Title/Abstract] OR "mouthrinse*"[Title/Abstract] OR "mouth rinse*"[Title/Abstract] OR
"crushed ice"[Title/Abstract] OR "slushie*"[Title/Abstract] OR "slushy*"[Title/Abstract]) ) AND (
("randomized controlled trial"[pt] OR "controlled clinical trial"[pt] OR randomized[Title/Abstract]
OR randomised[Title/Abstract] OR placebo[Title/Abstract] OR "drug therapy"[sh] OR
randomly[Title/Abstract] OR trial[Title/Abstract] OR groups[Title/Abstract]) NOT (animals[mh] NOT
humans[mh]) )
```

S1.3 EMBASE (via Ovid) — searched May 12, 2026

Using the MEDLINE (PubMed) strategy in Section S1.1 as the basis, the search was translated into the Ovid syntax using EMTREE thesaurus terms (exp = exploded thesaurus term; .tw. = text word; .pt. = publication type; .fs. = floating subheading; * = truncation). The complete strategy as executed is reproduced below. Records retrieved and exported for screening: n = 2,725.

```
(exp "surgical procedures, operative"/ OR surgery.tw. OR surgical.tw. OR operative.tw. OR
operation*.tw.) AND (((exp Ice/ OR Ice.tw. OR "cold stimul*".tw. OR exp Menthol/ OR exp Mouthwashes/
OR cooling.tw. OR cooled.tw. OR chilled.tw. OR refrigerat*.tw. OR iced.tw. OR menthol*.tw. OR
mentholated.tw. OR peppermint.tw. OR TRPM8.tw. OR "cooling agent*".tw. OR gargl*.tw. OR spray.tw. OR
swab*.tw. OR gauze.tw.) AND (oral.tw. OR mouth.tw. OR oropharyn*.tw. OR throat.tw. OR moisturis*.tw.
OR moisturiz*.tw. OR "lip hydrat*".tw.)) OR ("ice chip*".tw. OR "ice pop*".tw. OR popsicle*.tw. OR
"mentholated popsicle*".tw. OR "ice lolly".tw. OR "ice lollies".tw. OR "ice cube*".tw. OR "ice
water".tw. OR "iced drink*".tw. OR "cold drink*".tw. OR "cool water".tw. OR "oral cryotherapy".tw. OR
"cold water spray".tw. OR "cold spray".tw. OR "mouth spray".tw. OR "throat spray".tw. OR
"oropharyngeal moisturis* program*".tw. OR "cold water".tw. OR "wet gauze".tw. OR "moistened
gauze".tw. OR "sponge swab*".tw. OR lozenge*.tw. OR mouthrinse*.tw. OR "mouth rinse*".tw. OR "crushed
ice".tw. OR "slushie*".tw. OR "slushy*".tw.)) AND (("randomized controlled trial".pt. OR "controlled
clinical trial".pt. OR randomized.tw. OR randomised.tw. OR placebo.tw. OR "Drug Therapy".fs. OR
randomly.tw. OR trial.tw. OR groups.tw.) NOT (exp animals/ NOT exp humans/))
```

S1.4 Cochrane Library, including CENTRAL (via Wiley) — searched May 12, 2026

The strategy was translated into the Cochrane Library (Wiley) syntax ([mh] = MeSH heading; [mh /DT] = floating subheading Drug Therapy; :ti,ab = title/abstract; :pt = publication type; NEXT = adjacency operator; * = truncation). All sections of the Cochrane Library, including the Cochrane Central Register of Controlled Trials (CENTRAL), were searched. The complete strategy as executed is reproduced below. Records retrieved and exported for screening: n = 1,586.

```
([mh "surgical procedures, operative"] OR surgery:ti,ab OR surgical:ti,ab OR operative:ti,ab OR
operation*:ti,ab) AND ((([mh Ice] OR Ice:ti,ab OR ("cold" NEXT stimul*):ti,ab OR [mh Menthol] OR [mh
Mouthwashes] OR cooling:ti,ab OR cooled:ti,ab OR chilled:ti,ab OR refrigerat*:ti,ab OR iced:ti,ab OR
menthol*:ti,ab OR mentholated:ti,ab OR peppermint:ti,ab OR TRPM8:ti,ab OR ("cooling" NEXT
agent*):ti,ab OR gargl*:ti,ab OR spray:ti,ab OR swab*:ti,ab OR gauze:ti,ab) AND (oral:ti,ab OR
mouth:ti,ab OR oropharyn*:ti,ab OR throat:ti,ab OR moisturis*:ti,ab OR moisturiz*:ti,ab OR ("lip"
NEXT hydrat*):ti,ab)) OR (("ice" NEXT chip*):ti,ab OR ("ice" NEXT pop*):ti,ab OR popsicle*:ti,ab OR
("mentholated" NEXT popsicle*):ti,ab OR "ice lolly":ti,ab OR "ice lollies":ti,ab OR ("ice" NEXT
cube*):ti,ab OR "ice water":ti,ab OR ("iced" NEXT drink*):ti,ab OR ("cold" NEXT drink*):ti,ab OR
"cool water":ti,ab OR "oral cryotherapy":ti,ab OR "cold water spray":ti,ab OR "cold spray":ti,ab OR
"mouth spray":ti,ab OR "throat spray":ti,ab OR ("oropharyngeal" NEXT moisturis* NEXT program*):ti,ab
OR "cold water":ti,ab OR "wet gauze":ti,ab OR "moistened gauze":ti,ab OR ("sponge" NEXT swab*):ti,ab
OR lozenge*:ti,ab OR mouthrinse*:ti,ab OR ("mouth" NEXT rinse*):ti,ab OR "crushed ice":ti,ab OR
slushie*:ti,ab OR slushy*:ti,ab)) AND (("randomized controlled trial":pt OR "controlled clinical
trial":pt OR randomized:ti,ab OR randomised:ti,ab OR placebo:ti,ab OR [mh /DT] OR randomly:ti,ab OR
trial:ti,ab OR groups:ti,ab) NOT ([mh animals] NOT [mh humans]))
```

S1.5 CINAHL Plus (via EBSCOhost) — searched May 12, 2026

The strategy was translated into the EBSCOhost syntax using CINAHL Subject Headings (MH = subject heading, with + denoting explosion; TI = title; AB = abstract; PT = publication type; * = truncation). The complete strategy as executed is reproduced below. Records retrieved and exported for screening: n = 265.

```
((MH "surgical procedures, operative+") OR (TI surgery OR AB surgery) OR (TI surgical OR AB surgical)
OR (TI operative OR AB operative) OR (TI operation* OR AB operation*)) AND ((((MH Ice+) OR (TI Ice OR
AB Ice) OR (TI "cold stimul*" OR AB "cold stimul*") OR (MH Menthol+) OR (MH Mouthwashes+) OR (TI
cooling OR AB cooling) OR (TI cooled OR AB cooled) OR (TI chilled OR AB chilled) OR (TI refrigerat*
OR AB refrigerat*) OR (TI iced OR AB iced) OR (TI menthol* OR AB menthol*) OR (TI mentholated OR AB
mentholated) OR (TI peppermint OR AB peppermint) OR (TI TRPM8 OR AB TRPM8) OR (TI "cooling agent*" OR
AB "cooling agent*") OR (TI gargl* OR AB gargl*) OR (TI spray OR AB spray) OR (TI swab* OR AB swab*)
OR (TI gauze OR AB gauze)) AND ((TI oral OR AB oral) OR (TI mouth OR AB mouth) OR (TI oropharyn* OR
AB oropharyn*) OR (TI throat OR AB throat) OR (TI moisturis* OR AB moisturis*) OR (TI moisturiz* OR
AB moisturiz*) OR (TI "lip hydrat*" OR AB "lip hydrat*"))) OR ((TI "ice chip*" OR AB "ice chip*") OR
(TI "ice pop*" OR AB "ice pop*") OR (TI popsicle* OR AB popsicle*) OR (TI "mentholated popsicle*" OR
AB "mentholated popsicle*") OR (TI "ice lolly" OR AB "ice lolly") OR (TI "ice lollies" OR AB "ice
lollies") OR (TI "ice cube*" OR AB "ice cube*") OR (TI "ice water" OR AB "ice water") OR (TI "iced
drink*" OR AB "iced drink*") OR (TI "cold drink*" OR AB "cold drink*") OR (TI "cool water" OR AB
"cool water") OR (TI "oral cryotherapy" OR AB "oral cryotherapy") OR (TI "cold water spray" OR AB
"cold water spray") OR (TI "cold spray" OR AB "cold spray") OR (TI "mouth spray" OR AB "mouth spray")
OR (TI "throat spray" OR AB "throat spray") OR (TI "oropharyngeal moisturis* program*" OR AB
"oropharyngeal moisturis* program*") OR (TI "cold water" OR AB "cold water") OR (TI "wet gauze" OR AB
"wet gauze") OR (TI "moistened gauze" OR AB "moistened gauze") OR (TI "sponge swab*" OR AB "sponge
swab*") OR (TI lozenge* OR AB lozenge*) OR (TI mouthrinse* OR AB mouthrinse*) OR (TI "mouth rinse*"
OR AB "mouth rinse*") OR (TI "crushed ice" OR AB "crushed ice") OR (TI slushie* OR AB slushie*) OR
(TI slushy* OR AB slushy*))) AND (((PT "randomized controlled trial") OR (PT "controlled clinical
trial") OR (TI randomized OR AB randomized) OR (TI randomised OR AB randomised) OR (TI placebo OR AB
placebo) OR "Drug Therapy" OR (TI randomly OR AB randomly) OR (TI trial OR AB trial) OR (TI groups OR
AB groups)) NOT ((MH animals+) NOT (MH humans+)))
```

S1.6 Clinical trial registries—searched May 12, 2026

The following clinical trial registries were searched through their public web interfaces on May 12, 2026, to identify ongoing, unpublished, or unreported studies. Because the full database search strategy could not be directly

reproduced across the registry interfaces, simplified search strategies were used according to the search functions available in each registry. No restrictions on registration date or language were applied. Where available, recruitment status was left unrestricted. Records identified through the registries were handled separately from the database records (Section S1.8).

(a) ClinicalTrials.gov (https://clinicaltrials.gov):

```
Search: (postoperative OR surgery OR surgical) AND ("ice chips" OR popsicle OR menthol OR "cold water"
OR "cold spray" OR "cold gauze" OR "crushed ice")
```

(b) WHO International Clinical Trials Registry Platform, ICTRP (https://trialsearch.who.int):

```
Search: (postoperative OR surgery OR surgical) AND ("ice chips" OR popsicle OR menthol OR "cold water"
OR "cold spray" OR "cold gauze" OR "crushed ice")
```

(c) EU Clinical Trials Register (https://www.clinicaltrialsregister.eu):

```
Free-text search: thirst, ice, menthol
```

S1.7 Restrictions

No restrictions on publication date or language were applied to any database. All records available from database inception to May 12, 2026 were considered.

S1.8 Records retrieved by source

Database	Records retrieved and exported for screening
MEDLINE (via PubMed)	1,332
Cochrane Library (via Wiley)	1,586
EMBASE (via Ovid)	2,725
CINAHL Plus (via EBSCOhost)	265
**Total records retrieved**	**5,908**

Records identified through the clinical trial registries were handled separately from the database records and are not included in the total above. The full flow of records, including the number of duplicates removed and the number of studies screened, assessed for eligibility, and included at each stage, will be reported in the PRISMA 2020 flow diagram of the completed review.
